# Multimodal Imaging for Diagnosis of Anomalous Coronary Artery With Subsequent Myocardial Infarction

**DOI:** 10.1016/j.jaccas.2021.06.003

**Published:** 2021-08-19

**Authors:** Muhummad Sohaib Nazir, Rebecca Preston, Amedeo Chiribiri, Nabeel Sheikh

**Affiliations:** a School of Biomedical Engineering and Imaging Sciences, King’s College London, United Kingdom; b Department of Cardiology, St Thomas’ Hospital, London, United Kingdom; c Department of Radiology, St Thomas’ Hospital, London, United Kingdom

**Keywords:** anomalous coronary artery, multimodal imaging, myocardial infarction, CTCA, computed tomography coronary angiography, CMR, cardiovascular magnetic resonance, ECG, electrocardiogram, LGE, late gadolinium enhancement, RCA, right coronary artery

## Abstract

Chest pain in young adults is not always benign, and clinical suspicion should prompt further investigations. Multimodal imaging with computed tomography coronary angiography and cardiovascular magnetic resonance can be used to identify anomalous coronary arteries, determine adverse imaging features, and guide subsequent clinical decision making. (**Level of Difficulty: Beginner.**)

## Central Illustration

**Figure:**
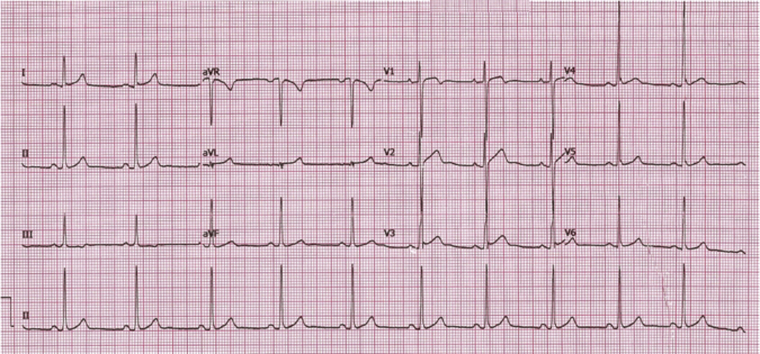

## History of Presentation

A 25-year-old man of African ethnicity was referred to a tertiary cardiac center following episodes of chest pain and collapse over a period of 2 years. He was previously investigated in another hospital where he underwent a transthoracic echocardiogram and 24-hour ambulatory Holter recording, both of which were normal. He was subsequently discharged from routine follow-up, but he continued to experience episodes of exertional chest pain, dizziness, and presyncope, predominantly while playing soccer, running quickly on the flat, or exerting himself on an incline. Of concern, he reported several episodes during soccer where he would develop chest pain followed by syncope while sprinting after the ball. As a result, he had deliberately reduced participation in exercise and sport to avoid developing these symptoms.

**Learning Objectives**

• To understand the role of multimodal imaging in the assessment of a young patient with chest pain.
• To appreciate the role of CTCA for detection of anomalous coronary arteries and CMR for assessment of myocardial scar using novel dark blood techniques.

## Past Medical History

He was usually fit and well with no past medical, drug, or family history of note, apart from his mother, who was diagnosed with hypertension. He denied cigarette smoking or recreational drug use.

## Differential Diagnosis

On the basis of the clinical presentation in a young patient, the potential differential diagnoses were valvular heart disease, hypertrophic obstructive cardiomyopathy, coronary artery disease, and noncardiac causes of chest pain.

## Investigations

**Figure 1:**
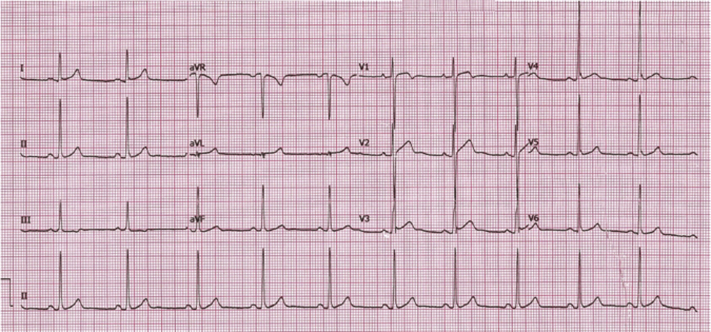
12-Lead Electrocardiogram Demonstrating Sinus Rhythm and Isolated Voltage Criteria for Left Ventricular Hypertrophy

**Figure 2:**
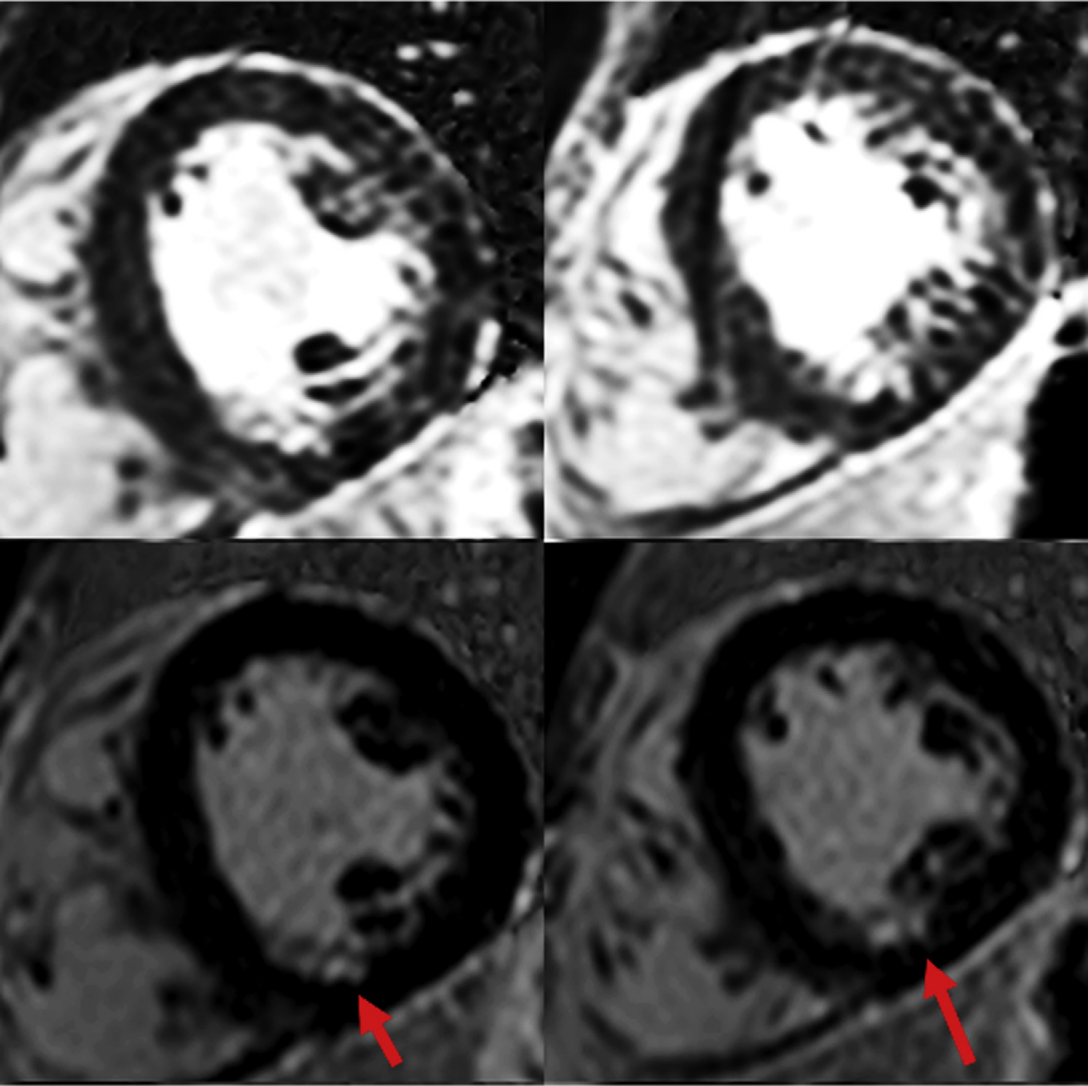
Phase-Sensitive Inversion Recovery Late Gadolinium Enhancement Images **(Top)** Standard “white blood” late gadolinium enhancement. **(Bottom)** “Dark blood” late gadolinium enhancement images. The small area of subendocardial myocardial enhancement **(arrows)** is better appreciated with dark blood images.

His clinical examination was unremarkable. He had normal blood pressure and resting heart rate of 60 beats/min. A 12-lead electrocardiogram (ECG) (Figure 1) confirmed sinus rhythm with isolated voltage criteria for left ventricular hypertrophy and no significant changes. To investigate the patient’s symptoms further, he underwent a cardiovascular magnetic resonance (CMR) scan, which demonstrated preserved left ventricular ejection fraction (58%), normal wall thickness (maximum 11 mm), and no evidence of left ventricular outflow obstruction (Videos 1, 2, 3, 4, and 5). There was hypokinesia of the midinferior wall (Video 6), which corresponded to an area of partial-thickness subendocardial myocardial enhancement on late gadolinium enhancement (LGE) imaging. The right ventricular size and function were normal, and there were no CMR features suggestive of an inherited cardiomyopathy. The extent and degree of myocardial infarction were better appreciated using novel dark blood imaging rather than standard LGE imaging (Figure 2).

**Figure 3:**
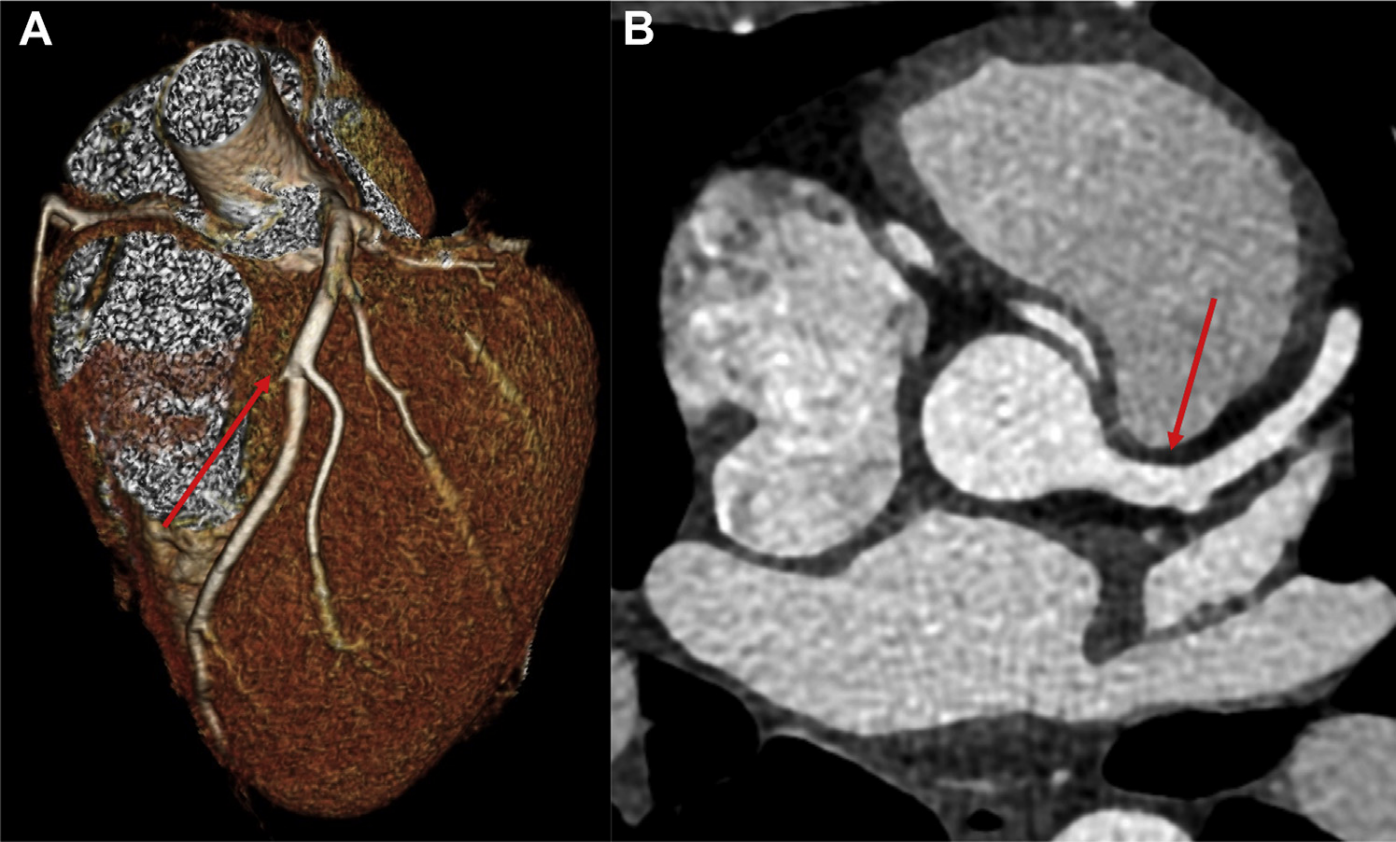
Normal Origin and Proximal Course of the Left Coronary Artery, Which Becomes the Left Anterior Descending Artery The left anterior descending artery **(arrows)** traverses the interventricular groove on **(A)** 3-dimensional volume-rendered reconstruction and **(B)** axial multiplanar reformats.

**Figure 4:**
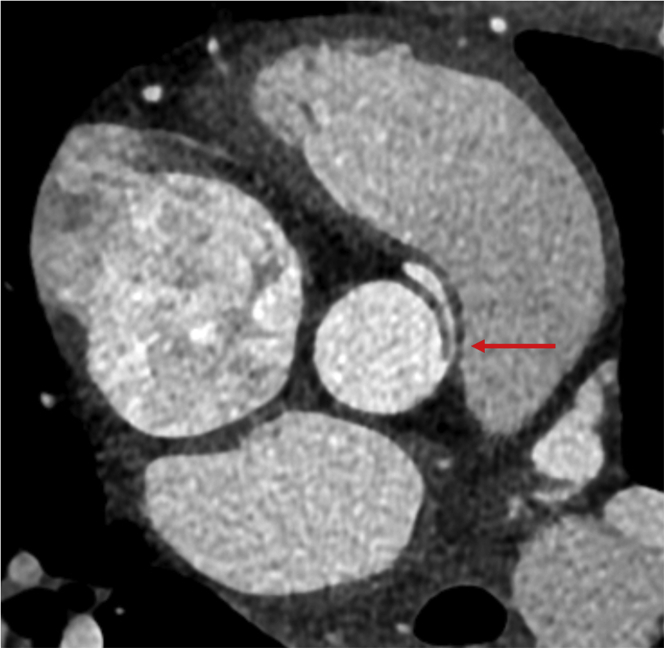
Computed Tomography Coronary Angiography Axial Multiplanar Reformat of the Right Coronary Artery Computed tomography coronary angiography demonstrates the slitlike, proximal intramural course of the right coronary artery, with an acute take-off **(arrow)**

**Figure 5:**
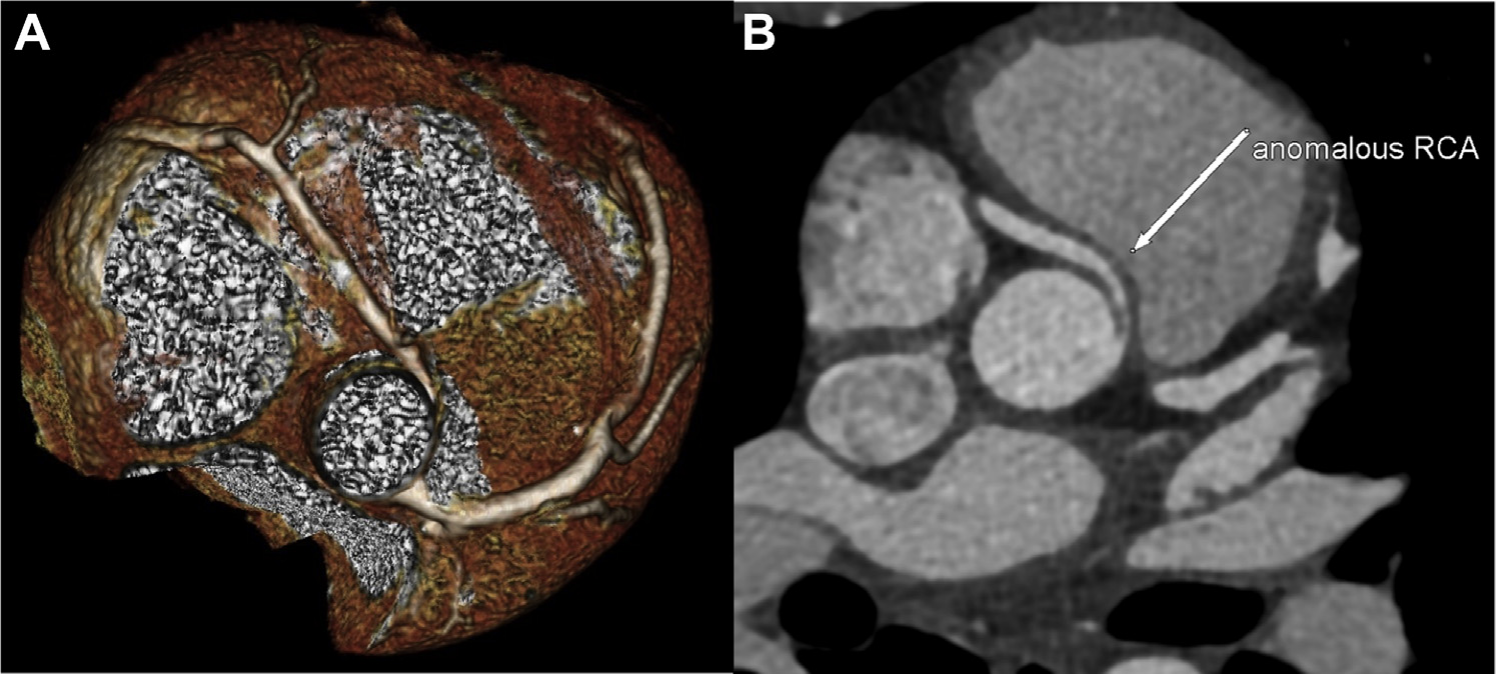
Computed Tomography Coronary Angiography of the Anomalous Right Coronary Artery **(A)** 3-dimensional volume-rendered and **(B)** axial multiplanar reformat images obtained from computed tomography coronary angiography of the anomalous right coronary artery, which follows a malignant interarterial course between the aorta and the pulmonary artery.

As a result of the CMR findings, computed tomography coronary angiography (CTCA) was performed. The origin and proximal course of the left coronary artery and left anterior descending artery were normal (Figures 3A and 3B), with no evidence of obstructive coronary artery disease in the left coronary system. However, the origin of the right coronary artery (RCA) was found to be anomalous. The RCA was found to arise from a separate coronary ostia arising from the left coronary sinus. The morphology of the proximal RCA was slitlike and followed an intramural course within the aortic wall, with an acute take-off angle of <45º (Figure 4). The proximal vessel then followed an interarterial course between the proximal aorta and the pulmonary artery (Figures 5A and 5B).

## Management

In light of his symptoms and imaging findings, our patient underwent elective surgery to correct the anomalous RCA by unroofing. A midline sternotomy incision and longitudinal pericardiotomy were made to expose the heart, and the patient was established on coronary bypass. Intraoperatively, on inspection, the RCA revealed a very small, slitlike origin and arose right next to the commissure of the left and right coronary leaflets. The intra-arterial course of the RCA was cannulated with a nerve hook, and the mural portion of the RCA was resected to a distance of at least 2 cm, until it arose from the middle of the right coronary sinus anteriorly. Prolene sutures were used to tack back the intima of the aorta to the opened-up roof of the RCA. This was performed at the level of the right coronary ostium anteriorly. At the end of the operation, a transesophageal echocardiogram confirmed a satisfactory position, demonstrating a normal, anteriorly orientated RCA.

## Discussion

Chest pain in a young person is not always benign, and syncope, especially when occurring *midexertion,* should always be investigated further to exclude underlying disease. According to most recent guidelines (1), this patient would be considered at low risk for coronary artery disease and would not warrant further anatomical or functional testing. Therefore, correct interpretation of the clinical history is crucial to avoid missing rarer causes of chest pain, such as in this patient.

Coronary anomalies are rare, with a reported incidence of approximately 1% (2). Anomalous coronary arteries with an interarterial course between the aorta and pulmonary arteries have the greatest potential for sudden cardiac death (3), and therefore timely diagnosis is highly relevant. We used multimodal imaging to determine the correct cause of our patient’s symptoms. CTCA has excellent spatial resolution and negative predictive value for coronary artery disease (4), although nascent methods and techniques with CMR that achieve isotropic high-resolution coronary images are emerging (5) and negate the need for radiation exposure in a young patient. Such CMR techniques still require future clinical evaluation.

Detection of subendocardial scar can be challenging with standard LGE imaging, particularly for small areas. The blood pool often has a high bright white signal intensity similar to the subendocardial scar, therefore potentially obscuring its detection. By using novel dark blood imaging with phase-sensitive inversion recovery images (6), better delineation of subendocardial scar can be achieved. This had significant clinical importance for our patient and ultimately confirmed the malignant nature of his anomalous RCA. There are sparse data on the impact of anomalous coronary arteries on myocardial function and coronary ischemia and how the use of a multimodal imaging approach with CTCA and CMR may aid in diagnosis and risk stratification. This should be an avenue of future research.

To our knowledge, this is the first time the impact of an anomalous coronary artery has been demonstrated with novel dark blood LGE imaging, and it demonstrates the valuable clinical utility of this novel technique alongside the importance of noninvasive multimodal imaging for diagnosis.

## Follow-Up

After the operation, the patient underwent assessment in the cardiac outpatient department, where he reported that his symptoms had completely resolved. He underwent an exercise treadmill test in which he performed 10 minutes of a Bruce protocol, achieving 96% of his age-predicted target heart rate and 12.6 METs of work without symptoms, ECG changes, or arrhythmias. He has since returned to his regular activities without further consequences.

## Funding Support and Author Disclosures

The authors acknowledge financial support from the Department of Health through the National Institute for Health Research (NIHR) comprehensive Biomedical Research Centre award to Guy’s & St Thomas’ NHS Foundation Trust in partnership with King’s College London and King’s College Hospital NHS Foundation Trust and by the NIHR MedTech Co-operative for Cardiovascular Disease at Guy’s and St Thomas’ NHS Foundation Trust. This work was supported by the Wellcome/EPSRC Centre for Medical Engineering (WT 203148/Z/16/Z). Dr Nazir has received funding from an NIHR Clinical Lecturership (CL-2019-17-001). The views expressed are those of the authors and not necessarily those of the DoH, the EPSRC, the NHS, the NIHR, or the Wellcome Trust. All other authors have reported that they have no relationships relevant to the contents of this paper to disclose.

## Appendix

Supplemental Video 1 4-Chamber Cine

Supplemental Video 2 2-Chamber Cine

Supplemental Video 3 3-Chamber Cine

Supplemental Video 4 Left Ventricular Outflow Tract Cine

Supplemental Video 5 Short-Axis Cine

Supplemental Video 6 Short-Axis Cine of Basal Inferior Wall Demonstrating Inferior Wall Motion Abnormality
